# Impact of Pre-Pregnancy Weight and Gestational Weight Gain on Birth Outcomes by Nativity in the United States: A Systematic Review

**DOI:** 10.3390/healthcare5040067

**Published:** 2017-09-29

**Authors:** Karen M. Tabb, Tumani Malinga, Maria Pineros-Leano, Flavia C. D. Andrade

**Affiliations:** 1 School of Social Work, University of Illinois at Urbana-Champaign, Urbana, IL 61801, USA; malinga2@illinois.edu (T.M.); pineros1@illinois.edu (M.P.-L.); 2 Department of Kinesiology and Community Health, College of Applied Health Sciences, University of Illinois at Urbana-Champaign, Champaign, IL 61820, USA; fandrade@illinois.edu

**Keywords:** nativity, pre-pregnancy weight, gestational weight gain, birth outcomes, systematic review

## Abstract

*Background*: Disparities in birth outcomes remain a problem in the United States. This study examined whether pre-pregnancy weight and gestational weight gain moderate the association between nativity and birth outcomes in the United States. *Methods*: We conducted a systematic review using Preferred Reporting Items for Systematic Reviews and Meta Analyses (PRISMA) guidelines. We searched PubMED, CINAHL, PsychInfo, and Cochrane Database of Systematic Reviews for relevant articles published before May 27, 2016. *Results*: Four articles met the eligibility criteria by adjusting for pre-pregnancy or gestational weight gain when examining birth outcomes by nativity. *Results*: Results from these studies show statistically significant differences in the risk of delivering low birth weight babies between foreign-born and U.S.-born women. These differences remained after adjusting for pre-pregnancy weight or gestational weight gain. However, results stratified by nativity still vary significantly by race/ethnicity. *Conclusion*: Few investigations include pre-pregnancy weight and gestational weight gain when examining differences in birth outcomes by nativity. Additional studies are needed to examine possible effect modification of these weight variables on the association between nativity and birth outcomes.

## 1. Introduction 

Gestational weight gain has attracted significant attention in recent years following the release of the 2009 Institute of Medicine guidelines for weight gain during pregnancy. Excessive gestational weight gain is associated with adverse birth outcomes, such as preterm birth, as well as negative outcomes for mothers, such as postpartum weight retention [1]. Past studies identify more favorable pregnancy outcomes for foreign-born (FB) women compared to U.S.-born women. Even though the Healthy Immigrant Effect posits that FB individuals often display better health than their U.S.-born counterparts, previous studies show that the relationship between race/ethnicity and nativity on birth outcomes is more complex [2,3]. To date, the bulk of research on disparities in birth outcomes has examined the role of behaviors, such as maternal overweight/obesity, or social characteristics, such as race/ethnicity, but few have examined the possible effect modification that pre-pregnancy and gestational weight gain can have on the association between nativity and birth outcomes.

In the U.S., nearly 25% of women ages 20 to 44 are obese [4], which presents a potential risk for reproductive health. Furthermore, 40–60% of women in the U.S. gain excessive weight during pregnancy [5,6]. The proportion is even higher among low-income racial/ethnic minorities—reaching a 62% gain in one study [7]. Women who are overweight or obese before getting pregnant are more likely to gain excessive weight during pregnancy [8]. This is particularly important because there are differences in obesity levels across race/ethnicity categories and nativity status among adult women [9]. In particular, compared to White women, U.S.-born Black women are more likely to be obese, whereas FB White and Hispanic women are less likely to be obese [9]. However, compared to White women, Black and Hispanic women are less likely to gain excessive weight during pregnancy [6]. These differences in pre-pregnancy weight and excessive gestational weight gain can help explain disparities in infant birth outcomes such as low birthweight (LBW) or preterm birth (PTB) [10,11]. Despite past studies on weight gain and birth outcomes, little is known about the moderating role of pre-pregnancy and gestational weight gain on the association between nativity and birth outcomes.

In the U.S., there are significant differences in PTB, LBW, and infant mortality rates (IMR) by nativity, race, and ethnicity [2,12]. Black mothers have higher rates of LBW and PTB than Whites, regardless of their nativity, whereas FB Latinas have PTB rates similar to U.S.-born Whites [2]. In addition, IMR are higher among U.S.-born White, Black, and Hispanics of Mexican heritage than their foreign-born counterparts [12]. A meta-analysis on ethnicity and preterm birth found that Black women were at greater risk for PTB [13]. Authors pointed out that the difference could be due to epigenetics; however, the review did not account for the critical factors of weight or nativity [13]. This systematic review aims to examine whether pre-pregnancy weight and gestational weight gain moderates the association between nativity and preterm birth and/or low birthweight outcomes in the U.S.

## 2. Materials and Methods

This systematic review used the Preferred Reporting Items for Systematic Reviews and Meta Analyses (PRISMA) approach. The current protocol was registered to the International Prospective Register of Systematic Reviews (PROSPERO) protocol under registration number CRD42016039146. Empirical quantitative studies conducted in the U.S. and published in peer-reviewed journals in English were included. Electronic databases, PubMED, CINAHL, PsychInfo, and Cochrane Database of Systematic Reviews, were searched for all available electronic articles published up to May 27, 2016.

Databases were searched using three search terms at a time to capture studies with any infant birth outcomes and gestational weight gain and distinctions of nativity or the pregnant woman’s birthplace. The term *pregnancy* was combined with *weight gain* (or *gestational weight*, or *body mass index*, or *weight*) and *nativity* (or *foreign born*, or *nationality*, or *ethnicity*) in all possible combinations.

Two research assistants independently conducted the initial search from all databases and combined search results using Endnote software libraries. All duplicate citations were removed. Titles and article abstracts were screened based on the following eligibility criteria: (1) reported birth outcomes; (2) provided detail on mother’s nativity; and (3) adjusted for pre-pregnancy weight or gestational weight gain. All full texts that met the eligibility criteria were reviewed by the lead author and research assistants, who discussed their relevance. We performed a quality assessment of the studies included, using the National Institutes of Health Quality Assessment Tool [14]. Articles were assigned a category of low, medium, or high, with lower scores representing higher risk of bias.

## 3. Result

**Figure 1:**
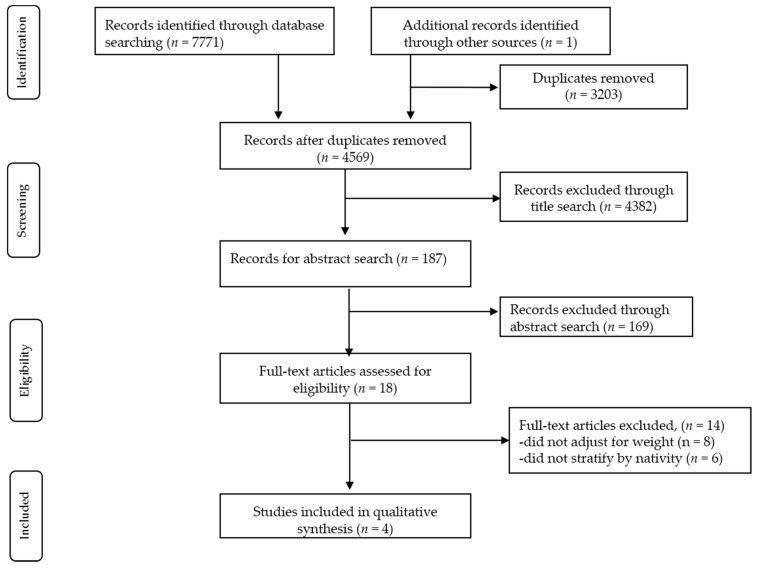
Identification of studies for inclusion in the systematic review.

The results of our search are shown in the PRISMA flow diagram (see Figure 1). A total of 7771 citations were retrieved from the original search. A total of 4532 articles were screened for eligibility after removing 3239 duplicates citations. After a title search, a total of 187 abstracts were reviewed, leaving 18 for full text review. Four studies met inclusion criteria [2,15,16,17]. The study findings and quality assessments are summarized in Table 1. Risk of bias assessment article scores ranged from 10 to 12. Quality score of 10 noted medium risk of bias and articles with a quality score of 11–12 were considered low risk of bias.

### 3.1. Study I

Almeida et al. [2] used the New York pregnancy risk assessment monitoring system (PRAMS) survey to examine the contribution of social ties and social support to the risk of LBW and PTB across women defined by race/ethnicity and nativity. The sample included 4443 women who gave birth between 2004 and 2007. Women who gained inadequate weight were nearly twice as likely to have LBW and PTB, but those who gained too much weight were less likely to have these negative birth outcomes than those with adequate weight based on the 1990 Institute of Medicine (IOM) Guidelines. This study did not explore possible interactions between nativity and gestational weight gain, but adjusted for pregnancy weight gain in all regression models.

### 3.2. Study II

Cabral et al. [15] used data from a single hospital in Boston in 1984. The sample included 616 U.S.-born and 201 FB Black women. Results indicate that FB Black women had heavier and lengthier infants at birth compared to U.S.-born Black women, even after controlling for weight gain during pregnancy. Compared to U.S.-born Black women, FB Black women had lower odds of LBW and PTB (OR 0.81, 95% CI 0.42–1.53 and 0.54, 95% CI 0.24–1.18, respectively), however, the differences were not statistically significant.

### 3.3. Study III

Flores et al. [16] examined a sample of 196,617 women from the Department of Health data on all births in Utah between January 2004 and December 2007. A higher proportion of U.S.-born Latina women were obese (21%) than their FB counterparts (15%) and U.S.-born White women (15%). In this sample, a higher proportion of U.S.-born Latinas gained more than appropriate weight during pregnancy (47%) than their FB counterparts (39%) and U.S.-born White women (43%). FB Latina women had a lower risk of LBW and PTB than U.S.-born Latinas, even after controlling for pre-pregnancy body mass index. Compared to U.S.-born White women, FB Latinas had a lower risk of PTB, but similar proportion of LBW infants (see Table 1).

### 3.4. Study IV

Hoggatt et al. [17] used birth records and survey data from Los Angeles County in 2003 including 2135 women. The major finding from this study was that Latina women had a higher prevalence of LBW compared to White women. However, higher LBW was found among low-acculturated FB Latinas who weighed less than 110 lbs. before pregnancy or had gained less than the recommended weight gain. Higher LBW was found among high-acculturated FB Latinas who weighed 200 lbs. or more before pregnancy, compared to U.S.-born Latinas. No statistical differences in LBW were found between U.S.-born and high-acculturated FB Latinas by gestational weight gain.

## 4. Discussion 

This systematic review sought to assess whether nativity-related differences in PTB and/or LBW were related to pre-pregnancy weight and gestational weight gain. We found four studies that examined the role of pre-pregnancy weight and weight gain during pregnancy on birth outcomes among different nativity groups. Although race/ethnicity remains a risk factor for health disparities, especially concerning PTB and LBW, much of the existing research has failed to include nativity of the mother when examining racial differences after taking into account pre-pregnancy weight and gestational weight gain. Past studies have shown that there is a significant amount of heterogeneity within the Black population as well as within the Hispanic population in the U.S., hence the need to examine the nativity status of the mothers. Even though the Healthy Immigrant Effect posits health advantages for FB individuals, results differed in studies that have explored the associations between pre-pregnancy weight, gestational weight gain, and birth outcomes. Past research finds that FB pregnant women tend to gain less weight during pregnancy than U.S.-born women [18,19].

Contrary to the Healthy Immigrant Effect, this review highlights mixed findings for birth outcomes by nativity when factoring in weight gain during pregnancy. For example, Flores et al. (2012) found that FB Latina women were less likely to be obese and to gain excessive weight than their U.S.-born counterparts [16]. FB Latina women were less likely to have LBW and PTB compared to their U.S.-born counterparts, even after controlling for pre-pregnancy body mass index [16]. Similarly, Cabral et al. (1990) showed that after adjusting for gestational weight gain, FB women were less likely to have LBW and PTB than their U.S.-born counterparts [15]. However, Hoggatt and colleagues (2012) did not find significant findings for differences in LBW by nativity among high-acculturated Latina and their U.S.-born counterparts after adjusting for gestational weight gain [17]. The findings from Hoggatt et al. [17] present limitations related to their unique sample of underweight women, which may speak to unmeasured risk factors such as food insecurity.

In this review, studies acknowledged that beyond nativity, it is important to explore how acculturation and length of stay in the U.S. can influence health outcomes among the FB population. Acculturation tends to be associated with changes in health behaviors, particularly the adoption of diets that are linked to weight gain, which increases the risk for worse birth outcomes [17]. Higher levels of acculturation are also associated with smoking. In addition, past studies found that maternal smoking is associated with increased weight changes [7]. Given the risk for adverse health behaviors to increase with acculturation, future studies should account for nativity, but also for measures of acculturation to better understand mechanisms for underlying disparities.

While there are many strengths to this systematic review, there are also some limitations. First, the scope of the search term *pregnancy* is broad and articles on gestational weight gain and postpartum health may not be included. Another limitation is the lack of reporting on gestational age (large or small for their gestational age at birth) across the four studies in this review. While past studies show that disparities such as gestational age are associated with pre-pregnancy weight and gestational weight gain, we are unable to address these associations based on the studies included in this review. Moreover, the search results included a 25-year span of research, which overlaps with several changing patterns in immigration in the U.S. During this time span, there was also a drastic increase in non-communicable diseases and obesity, which might contribute to poorer birth outcomes. Finally, during the 25-year time frame, the IOM guidelines were updated, but the sampling frames and comparisons do not reflect the most up-to-date guidelines. Therefore, the results summarized in this review are descriptive and cannot be used for making direct statistical comparisons with studies using the 2009 IOM guidelines. Future studies are needed to monitor the associations between nativity status, pre-pregnancy weight and gestational weight gain, and birth outcomes. Even though this review sought to evaluate whether nativity-related differences in PTB and LBW might be related to pre-pregnancy weight and gestational weight gain, the small number of studies limits our conclusions. Additional studies are necessary to better understand whether pre-pregnancy weight and gestational weight gain can help explain some of the nativity differences in birth outcomes. This is particularly important given the changes in obesity prevalence in the U.S. and abroad, as well as changes in immigration patterns.

## 5. Conclusions

Even though this review sought to evaluate whether nativity-related differences in PTB and LBW might be related to pre-pregnancy weight and gestational weight gain, the small number of studies limits our conclusions. Additional studies are necessary to better understand whether pre-pregnancy weight and gestational weight gain can help explain some of the nativity differences in birth outcomes. This is particularly important given the changes in obesity prevalence in the U.S. and abroad, as well as changes in immigration patterns.

## Figures and Tables

**Table 1:** Characteristics and results from studies on birth outcomes by nativity measuring weight gain during pregnancy.

Article Reference Number	Sample Size *	Race/Ethnicity	Low Birth Weight OR (95% CI)		Preterm Birth OR (95% CI)		Weight Gain	Bias Risk
			Foreign-Born	U.S.-Born	Foreign-Born	U.S.-Born		
2	*N* = 1128: USB = 757, FB = 371	Non-Latina White	0.79 (0.56, 1.12)	1.0	0.47 (0.26, 0.84)	1.0	Controlled for weight gain during pregnancy	Low
2	*N* = 1231: USB = 676, FB = 555	Non-Latina Black	2.24 (1.68, 2.99)	2.52 (1.90, 3.33)	2.60 (1.60, 4.24)	2.43 (1.56, 3.77)	Controlled for weight gain during pregnancy	Low
2	*N* = 499: USB = 52, FB = 447	Asian	n/a	1.47 (1.08, 2.00)	n/a	1.23 (0.71–2.14)	Controlled for weight gain during pregnancy	Low
2	*N* = 371: USB = 285, FB = 86	Puerto Rican (Island/Mainland)	2.07 (1.17, 3.67)	2.04 (1.43, 2.91)	1.72 (0.73, 4.06)	1.17 (0.66, 2.09)	Controlled for weight gain during pregnancy	Low
2	*N* = 1,214: USB = 233, FB = 981	Other Latina	1.35 (1.02, 1.78)	1.44 (0.98, 2.12)	1.53 (0.95, 2.49)	1.84 (0.99, 3.41)	Controlled for weight gain during pregnancy	Low
15	*N* = 817: USB Black (ref) 616, FB Black 201	Black	0.81 (0.42, 1.53)	1.0	0.54 (0.24, 1.18)	1.0	Controlled for weight gain during pregnancy	Medium
16	*N* = 196,617: White (ref) 164,690, USB Latina 10,122, FB Latina 21,805	White, Latina	0.95 (0.88, 1.03)	1.14 (1.05, 1.25)	0.85 (0.79–0.91)	1.04 (0.96–1.12)	Controlled for pre-pregnancy body mass index	Medium
17	*N* = 2135: White (ref) 431, USB Latina 495, FB Latina 1209	White, Latina	1.32 (1.18, 1.49)	1.34 (1.17, 1.53)			Controlled for pregnancy weight gain	Low

* USB stands for U.S.-born and FB stands for foreign-born.
